# Medications for opioid use disorder during incarceration and post-release outcomes

**DOI:** 10.1186/s40352-023-00209-w

**Published:** 2023-02-04

**Authors:** Lara Cates, Aaron R. Brown

**Affiliations:** 1 Department of Social Work, Western Carolina University, 3971 Little Savannah Road, Cullowhee, NC 28723 USA; 2 College of Social Work, University of Kentucky, 619 Patterson Office Tower, Lexington, KY 40506 USA

**Keywords:** Opioid, Substance use disorder, Incarceration, Recidivism, Overdose

## Abstract

**Background:**

Continuation or initiation of MOUDs during incarceration could improve post-release outcomes by preventing return to opioid use and reducing risk of overdose. People with OUD involved in the criminal legal system are a vulnerable population, yet little research has comprehensively examined post-release outcomes associated with receiving MOUDs in jail and prison settings.

**Methods:**

The authors conducted a review of published peer-reviewed literature on post-release outcomes associated with the use of MOUDs in correctional settings to determine implications for further research and policy.

**Results:**

Results showed compelling evidence supporting the use of MOUDs for currently incarcerated populations, with almost all studies showing that MOUDs provided during incarceration increased community-based treatment engagement post-release. There is also evidence that initiating or continuing MOUDs during incarceration is associated with decreased opioid use and overdoses post-release, without increasing criminal involvement.

**Conclusions:**

Findings indicate that forcing tapering and withdrawal during incarceration can have dire consequences upon release into the community. Initiating or continuing MOUDs during incarceration reduces the risk for opioid use and overdose upon release by maintaining opioid tolerance and increasing community treatment engagement.

## Highlights

- Providing medications for opioid use disorder to incarcerated populations is associated with increased engagement in community-based treatment post-release.
- There is strong evidence to support the adoption of MOUDs in correctional institutes due to their association with decreased opioid use, injection drug use, and overdoses after release from incarceration.
- Findings indicate that forcing tapering and withdrawal during incarceration can increase risk of overdose and death upon release into the community.

## Introduction

The rise of opioid-related overdoses in the United States is a public health crisis that has gained much attention in recent years, and vital statistics that have been released since the COVID-19 pandemic are sobering. Between April 2020 and April 2021, there were an estimated 75,673 opioid-related overdose deaths in the United States, an increase of 35% from the same period the year before (Centers for Disease Control and Prevention Centers for Disease Control and Prevention, 2021). Along with the opioid crisis, the United States has been experiencing a crisis of incarceration. The U.S. has the highest rate of incarceration in the world, with almost 2.1 million people held in correctional facilities (Minton et al., 2021). Rates of opioid use among those involved in the criminal legal system are disproportionately high compared to the general population in the U.S., and those who have been recently released from prison are at increased risk of drug overdose death (Binswanger et al., 2013; Merrall et al., 2010; Mumola et al., 2007). This is an issue that must be understood within the context of the U.S. legal system which has aggressively enforced the criminalization of drug use for at least half a century, leading to the mass incarceration of people who use drugs (PWUD) and those with substance use disorders (SUDs; Pew, 2018). The vast majority (85%) of the prison population in the U.S. have drug-related convictions and/or have an active SUD (National Institute on Drug Abuse, 2020). People who inject drugs have a higher rate of recidivism, largely due to the criminalization of the drugs people typically inject (Håkansson & Berglund, 2012).

People with opioid use disorder (OUD) involved in the criminal legal system are at a higher risk for opioid-related overdose due to many factors including decreased tolerance after forced withdrawal while incarcerated, insufficient counseling prior to release, correctional facilities’ failure to recognize individuals who are at risk for return to use, and lack of post-release follow-up (Binswanger et al., 2013; Møller et al., 2010). Upon release from incarceration, many return to opioid use, and two-thirds are rearrested for a new offense within 3 years (de Andrade et al., 2018; Langan & Levin, 2002). Inadequate social support, poverty, stigma, and other barriers to accessing medications for opioid use disorder (MOUDs) in the community further increase the risk of overdose post-release (Joudrey et al., 2019).

Methadone, buprenorphine, and naltrexone are the MOUDs that are currently approved by the Food and Drug Administration (FDA) for treatment of OUD (Food and Drug Administration, 2019). The use of MOUDs is considered the gold standard, evidence-based treatment for OUD, although there is stronger evidence for the effectiveness of agonists than for naltrexone (National Academies of Sciences, Engineering, and Medicine, 2019). MOUDs significantly increase an individual’s engagement in treatment and reduce illegal opioid use compared to non-pharmacological approaches (Comer et al., 2006; Fudala et al., 2003; Mattick et al., 2009). Participation in agonist-based MOUDs is associated with reductions in the risk for all-cause and overdose mortality (Sordo et al., 2017).

While there is compelling research regarding the effectiveness of MOUDs, they are not commonly provided to people with OUD who are housed in correctional settings (Nunn et al., 2009; Scott et al., 2021). Among the small number of correctional facilities that have adopted MOUDs, methadone is typically the only medication provided, and it is often only available to specific populations such as pregnant women or people with chronic pain (Fiscella et al., 2004; Nunn et al., 2009). Consequently, people with OUD regularly endure forced withdrawal from opioids, including MOUDs, upon incarceration. Not only is forced withdrawal extremely uncomfortable to endure but it is also associated with an increased risk of opioid-related overdose after release (Degenhardt et al., 2014; D'Hotman et al., 2019). Furthermore, the ubiquity of forced withdrawal during incarceration affects the utilization of MOUDs in the community as PWUD are less likely to initiate MOUDs for fear of losing access during incarceration and undergoing severe withdrawal symptoms again (Maradiaga et al., 2016). Continuation or initiation of MOUDs during incarceration could increase utilization of this life-saving treatment, aid in preventing the return to illegal opioid use, prevent overdose post-release, and mitigate other risks of opioid use such as the spread of infectious diseases and recidivism.

People with OUD involved in the criminal legal system are a vulnerable population, yet little research has comprehensively examined the post-release outcomes associated with the use of MOUDs in jail and prison settings. People with OUD who are incarcerated must have access to effective, evidence-based treatment during their incarceration and before being released back into the community. This review aims to examine the extant peer-reviewed literature on post-release outcomes associated with the use of MOUDs in prison and jail settings in the United States and to determine implications for further research and policy.

## Method

A review of published, peer-reviewed literature was conducted by the authors using PsycINFO, PubMed, and Web of Science databases. No date restrictions were used apart from the search end date of September 20, 2022. Both databases were searched using the following terms: *(jail OR prison OR incarceration OR incarcerated)* and *(post-release OR post release)* and *(“medication for opioid use disorder” OR MOUD OR “medication assisted treatment” OR MAT OR “methadone maintenance therapy” OR MMT OR buprenorphine OR methadone OR suboxone OR naltrexone)* and (*outcomes)*. To identify additional articles specifically related to the implementation of naltrexone during incarceration, a second search was conducted with the same databases using the following terms: *(jail or prison or incarceration or incarcerated)* and *(naltrexone OR vivitrol OR XR-NTX)*. The following criteria were used to select articles for inclusion: 1) empirical study; 2) sampled participants who had been incarcerated in the United States; 3) examined post-release outcomes associated with the use of MOUD while incarcerated; 4) peer-reviewed; 5) published in English; 6) peer-reviewed journal article; and 7) published prior to September 20, 2022. Articles were excluded if they only examined the use of MOUDs during incarceration for pharmacologically-assisted withdrawal.

**Fig. 1:**
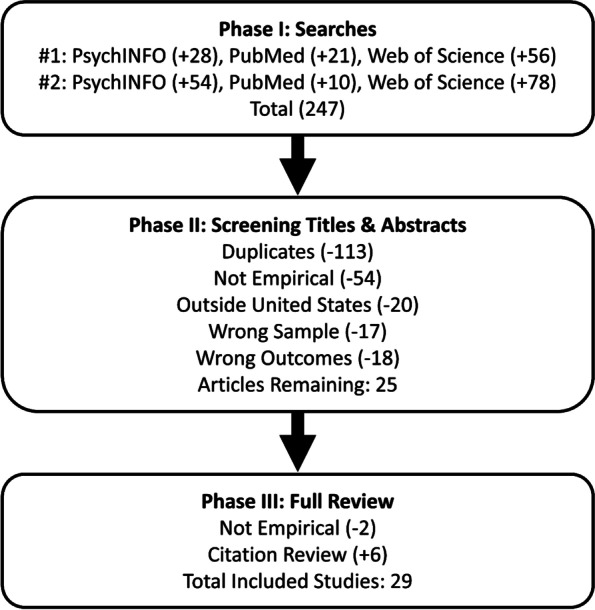
Search and Distillation

Both authors participated in the distillation and review of search results. Each author separately distilled the results during each phase and then met to reach agreement on the final inclusion of articles for the review. The searches produced 134 unique articles, after removing duplicates (see Fig. 1). Titles and abstracts were first reviewed to select potentially eligible articles, and the potentially eligible articles were then retrieved for full review to further assess for inclusion criteria and to gather results from articles that met criteria. The citations from articles included in the full review phase were screened for articles that met inclusion criteria but were not found in searches. After reviewing titles and abstracts, 25 articles remained. During full review of these 25 articles, two were excluded for not meeting criteria and six additional eligible articles were identified among citations of the reviewed articles leading to a total of 29 articles included in this review. The most common reasons for ineligibility were: 1) did not include empirical data, 2) were conducted outside the United States, 3) assessed only the feasibility of cost for providing MOUD in correctional settings, and 4) focused on MOUD treatment post-release rather than while incarcerated. See Fig. 1 for a diagram of the distillation process. Both authors separately reviewed and coded all 29 included articles for findings, conclusions, limitations, and the use of rigorous methodological procedures such as probability sampling, comparison groups, randomization to groups, biological verification of substance use, follow-up intervals, and intent-to-treat analyses. After independently coding each article, the authors met to reach agreement regarding the results, limitations, and implications of the included studies.

## Results

### Characteristics of included studies

The 29 included articles reported results from 22 distinct studies (see Table 1). Several of the studies published results across multiple articles, differentiated only by follow-up intervals and outcomes (e.g., Gordon et al., 2008; Kinlock et al., 2007; Kinlock et al., 2009). Among the 22 studies, half (11 of 22) implemented between-groups designs with randomization to at least one treatment group and either a control group (e.g. treatment-as-usual) or a comparison group (e.g. counseling-only). The other eleven studies implemented various non-experimental designs including retrospective case-control, quasi-experimental, and observational methodologies. The mean sample size among the included studies was 777 (*SD* = 3008; Skewness = 5.31), with a median of 200 and ranging from 15 to 16,349. Most studies utilized non-probability sampling methods with the exceptions of the Dole et al. (1969) study that used random selection and the Magura et al. (1993) study which used systematic sampling. Studies examined various types of MOUDs: nine looked at methadone only, three included buprenorphine only (including one study of buprenorphine/naloxone), six examined time-released naltrexone (XR-NTX) only, one looked at levo-alpha acetylmethadol (LAAM), and three examined more than one medication. Most of the studies took place in the Northeastern and Mid-Atlantic regions of the United States, and seven states were represented across the studies, listed here in order of how many studies were conducted in each state: New York (6), Maryland (5), Rhode Island (5), Connecticut (2), Massachusetts (2), New Mexico (1), and Pennsylvania (1). Most of the studies (13 of 22) examined people who had been incarcerated in jails only, while five studies examined those in prison, and four included samples of people incarcerated in either jails or prisons.

**Table 1:** Included Articles Grouped by Study/Trial in Chronological Order

Citations	Sample size	Setting	Post-release follow-up	Medications & comparisons	Findings
Dole et al., (1969)	28	New York, NY Jail	Weekly for 7–10 months	Methadone vs. no medication	Compared a control group, those in the methadone group used opioids less on average and were less likely to have additional convictions during the post-release follow-up period.
Magura et al., (1993)	446	New York, NY Jail	1 month 5 months	Methadone vs. no medication	Participants who received methadone while incarcerated were more likely to enter and remain in community treatment than control participants at 1-month and 5-months post-release.
Tomasino et al., (2001)	16,349	New York, NY Jail	11 years	Methadone (no comparison)	Those receiving methadone while incarcerated reported to community based treatment 74–80% of the time over a 5 year period. Those who did not have a history of SUD treatment prior to their incarceration were less likely to continue treatment in the community after release. Recidivism among participants was low during an 11-year monitoring period.
Kinlock et al., (2005)	64	Baltimore, MD Prison	6 months 9 months	LAAM vs. referral only	Those who received LAAM while incarcerated were more likely to enter community treatment and remain in treatment at follow-up than those who did not receive LAAM. LAAM participants also reported less crime-related income at follow-up.
Kinlock et al., (2007)^*^; Gordon et l., (2008)^*^; Kinlock et al., (2009)^*^	204	Baltimore, MD Prison	1 month 6 months 12 months	Methadone + counseling vs. counseling + transfer vs. counseling + referral	The methadone plus counseling group was more likely to enter community treatment compared to the other groups. The methadone plus counseling group was more likely to remain in treatment and have opioid-negative urine specimens at 1-, 6-, and 12-months post-release compared to the other groups. Those in the methadone condition spent more days in treatment post-release compared to the other groups. The methadone plus counseling group was less likely to report criminal activity at 6-months post-release but arrest records at 12-months showed no differences between groups.
Magura et al., (2009)	116	New York, NY Jail	3 months	Methadone vs. buprenorphine	Those receiving buprenorphine while incarcerated were less likely to withdraw voluntarily from medication while incarcerated than those who received methadone. Those who received buprenorphine while incarcerated were more likely to continue treatment in the community and report intentions to remain in treatment than those who received methadone while incarcerated.
McKenzie et al., (2012)^*^	62	Rhode Island Department of Corrections Jail & Prison	12 months	Methadone vs. referral with financial assistance vs. referral only	Those who received methadone prior to release were more likely to enter community treatment after release and to do so in a shorter time compared to the other groups. Among those who did enter community treatment, those who received methadone pre-release reported less opioid use and injection drug use at 6-months post-release than the other groups
Zaller et al., (2013)	44	Rhode Island Department of Corrections Jail & Prison	9 months	Buprenorphine/naloxone pre-release vs. post-release	Those who initiated buprenorphine/naloxone prior to release reported less heroin use and were more likely to remain in treatment 6-months post-release compared to those who initiated post-release.
Gordon et al., (2014)^*^; Gordon et al., (2017)^*^; Gordon et al., (2018)^*^	211	Baltimore, MD Prison	12 months	Buprenorphine + counseling vs. counseling only	Receiving buprenorphine + counseling while incarcerated led to a higher likelihood of entering community treatment compared to counseling only. Those who received buprenorphine prior to release engaged in community treatment more days during follow-up compared to those who received counseling only. There was not a significant effect on arrests or crime severity post-release.
Gordon et al., (2015)	27	Baltimore, MD Prison	6 months	At least 6 injections of XR-NTX vs. fewer	Those who completed at least 6 injections were less likely to use opioids or cocaine than those who had fewer injections. There were no significant differences for re-arrests or re-incarceration.
Lee et al., (2015)^*^	34	New York, NY Jail	1 month	XR-NTX vs. no medication	Those who received XR-NTX prior to release were less likely to relapse to opioid use at 1-month post-release compared to those who did not receive medication prior to release.
Rich et al., (2015)^*^; Brinkley-Rubenstein et al., (2018)^*^	223	Rhode Island Department of Corrections Jail & Prison	12 months	Methadone vs. forced taper/withdrawal	Those who continued methadone while incarcerated were more likely to re-engage in community treatment and less likely to report opioid use at 1-month post-release compared to those who were forced to taper and withdraw while incarcerated. Those who were on methadone the entire time they were incarcerated reported fewer non-fatal overdoses and were less likely to report heroin use or injection drug use at 12-months post-release compared to those who were not receiving methadone immediately prior to their release.
Friedman et al., (2018)^*^	15	Rhode Island Department of Corrections Jail & Prison	18 months	Pre-release vs. post-release initiation of XR-NTX	The pre-release group had more days of confirmed abstinence from opioid use during the first month post-release. Time to relapse was longer for the pre-release group as well. Only 17% of post-release group received more than 1 injection compared to 78% in the pre-release group.
Green et al., (2018)	336	Rhode Island Department of Corrections Jail & Prison	6 months	Buprenorphine, methadone, or XR-NTX vs. no medication	There was a 60.5% reduction in mortality amongst recently incarcerated individuals due to overdose deaths after the implementation of a state-wide program which continued MOUDs during incarceration.
Lincoln et al., (2018)	67	Hampden County, MA	6 months	Pre-release vs. post-release initiation of XR-NTX	Treatment retention was higher throughout the follow-up period for the pre-release group. There were 3 overdose deaths, all among the pre-release group after stopping XR-NTX
Moore et al., (2018)	382	Connecticut Department of Corrections Jail	6 months	Methadone vs. forced taper/withdrawal	Continuing methadone during incarceration increased odds of re-engaging in treatment post-release compared to forced taper and withdrawal. Those who received methadone from the same provide prior to, during, and after incarceration were less likely to recidivate.
Velasquez et al., (2019)^a^	33	New York, NY Jail	1 week – 19 months (*M* = 3.5 months)	XR-NTX, methadone, or buprenorphine vs. no medication	Most had never heard of XR-NTX and were skeptical of the effectiveness XR-NTX’s blockade effects, however most were satisfied with XR-NTX once they took it. Discontinuation of XR-NTX was attributed to high exposure to drug-using peers. Those who took methadone or buprenorphine were also satisfied with the treatments, although those on methadone reported dissatisfaction with daily observed dosing. Unstable housing and economic insecurity were identified barriers to treatment engagement.
Farabee et al., (2020)^*^	135	Albuquerque, NM Jail	12 months	XT-NTX + PN vs. XR-NTX vs. ETAU	The XR-NTX + PN group reported less opioid use and sex-related HIV risk at 12-months post-release compared to the ETAU group.
Kelly et al. (2020)^*^; Schwartz et al., (2020)^*^; Schwartz et al., (2021)^*^	225	Baltimore, MD Jail	24 months	Methadone + PN vs. methadone vs. ETAU	Those who received interim methadone (with or without PN) during pre-trial detention were more likely to enter community treatment upon release and to remain in treatment at 1-, 3-, and 6-months post-release than those who did not receive methadone. There were no significant differences between groups in treatment engagement, opioid use, likelihood of arrest, or crime severity at 12-months or 24-months post-release.
Haas et al., (2021)	1564	New Haven and Bridgeport, CT Jail	5 days – 63 months (*M* = 16 months)^b^	Methadone vs. forced taper/withdrawal	Continuation of methadone throughout incarceration was associated with a significant decrease in non-fatal overdoses and a greater likelihood of resuming methadone treatment in the community post-release. Those who resumed methadone in the community had lower odds of overdose death compared to those who did not.
Woody et al., (2021)^*^	86	Philadelphia, PA Jail	6 months	Pre-release vs. post-release initiation of XR-NTX	Treatment adherence was higher for those who initiated XR-NTX before release. There were no differences in relapse between groups. There were fewer overdoses during follow-up among the pre-release group.
Evans et al., (2022)	469	Franklin and Hampshire Counties, MA Jail	*M* = 23 months^*^	Buprenorphine vs. no medication	Those receiving buprenorphine while incarcerated were less likely to recidivate compared to those who did not receive buprenorphine.

*LAAM* levo-alpha-acetylmethadol, *XR-NTX* extended-release naltrexone, *PN* patient navigation, *ETAU* enhanced treatment as usual

^*^Randomized Controlled Trial

^a^qualitative study

^b^used multiple secondary data sources for outcomes

### Community-based treatment engagement

#### Methadone

Several studies examined the effect of methadone on post-release community-based treatment engagement and found similar results. Magura et al. (1993) found that participants in a jail methadone treatment program entered and remained in community-based treatment at higher rates than non-participants. Haas et al. (2021) found that participants who received methadone treatment while incarcerated had higher rates of continuing methadone treatment in the community, and McKenzie et al. (2012) found that participants who started methadone treatment prior to release were significantly more likely to enter community-based treatment post-release. Moore et al. (2018) found that participants who continued methadone treatment while incarcerated were more likely to engage with community-based methadone treatment provider upon release. Brinkley-Rubinstein et al. (2018) found that participants who received methadone treatment prior to release from incarceration were significantly more likely than participants who did not receive methadone treatment prior to release to continuously engage in methadone treatment in the community during the 12-month follow-up period. Another study found that participants who received methadone treatment while incarcerated were significantly more likely than a counseling-only group to enter and continue in community-based treatment at 1-, 6-, and 12-months post-release (Gordon et al., 2008; Kinlock et al., 2007; Kinlock et al., 2009). A study by Rich et al. (2015) found that participants who were allowed to continue methadone treatment while incarcerated were two times as likely to return to community-based treatment within one month of release compared to participants who endured forced withdrawal. However, Schwartz et al. (2020) found that while significantly more participants in the methadone groups engaged in treatment compared to the treatment-as-usual group at 1-month post-release, there were no significant differences between groups in treatment engagement at 12-months post-release.

Tomasino et al. (2001) did not have a comparison group for the Key Extended Entry Program (KEEP) at Rikers Island, however they found that those who initiated or continued methadone during incarceration reported to community-based treatment 74% to 80% of the time over a 5-year program period. Prior treatment history was associated with reporting to community-based treatment, such that those who did not have a history of substance disorder prior to their incarceration were less likely to continue treatment in the community after release.

#### Buprenorphine

Several studies examined the effect of buprenorphine during incarceration on post-release community-based treatment engagement. One study found that participants who initiated buprenorphine treatment while incarcerated were more likely to enter community treatment soon after release and had spent more days in community treatment at 12-months post-release compared to those who initiated buprenorphine after release (Gordon et al., 2014; Gordon et al., 2017). Similarly, Zaller et al. (2013) found that participants who initiated buprenorphine/naloxone treatment while incarcerated entered community treatment in fewer days and engaged in treatment for longer periods compared to participants who initiated treatment post-release. A study by Magura et al. (2009) compared buprenorphine to methadone and found that participants who received buprenorphine treatment while incarcerated reported to post-release community treatment significantly more often than participants who received methadone treatment while incarcerated.

#### Naltrexone

Studies examining XR-NTX generally saw low rates of treatment participation after 1–2 months post-release. Farabee et al. (2020) found that 36% of participants who received XR-NTX plus patient navigation prior to release attended at least one post-release session and treatment retention was low throughout the follow-up period. Lee et al. (2015) found no significant difference in rates of community substance use treatment engagement when comparing those who received XR-NTX prior to release to those who received no medication, although 75% of participants in the naltrexone group received a second XR-NTX injection 1-month post-release, suggesting high levels of short-term post-release engagement for XR-NTX. Similarly, in a study by Gordon et al. (2015), 78% of the 27 participants who received XR-NTX prior to release received an injection 1 month after release, however only 10 (37%) completed all six monthly injections that were offered after release. Friedmann et al. (2018) found that 78% of those who received XR-NTX prior to release received more than one injection compared to 17% of those who started after release, and those in the pre-release XR-NTX group attended more treatment appointments post-release (46% vs. 22%). Lincoln et al. (2018) and Woody et al. (2021) also saw higher community treatment retention among those who started XR-NTX pre-release compared to those who started after release. In their qualitative study on barriers and experiences with MOUDs following release from jail, Velasquez et al. (2019) found that some participants stopped taking XR-NTX due to continued exposure to drug-using peers and a desire to feel the euphoric effects of illegal opioids.

#### Levo-alpha Acetylmethadol

In the only study of LAAM, Kinlock et al. (2005) found substantial differences between groups, with 95% of participants who received LAAM entering community-based treatment compared to 10% of the control group and nearly 50% of those in the LAAM group being retained in treatment at 6-months.

### Opioid use

#### Methadone

Several studies examined the effect of methadone treatment during incarceration on post-release opioid use, generally finding that initiating or continuing methadone during incarceration was associated with reduced opioid use and injection drug use upon release. One study found that participants who continued methadone treatment while incarcerated were significantly less likely to report past 30-days heroin use and injection drug use at 12-months post-release compared to those who were forced to taper and withdraw during incarceration (Brinkley-Rubinstein et al., 2018; Rich et al., 2015). Another study found that participants who received methadone treatment and counseling while incarcerated were significantly less likely than a counseling-only group to have an opioid-positive urine sample at 1-, 6-, and 12-months post-release (Gordon et al., 2008; Kinlock et al., 2007; Kinlock et al., 2009). A study conducted by McKenzie et al. (2012) found that participants who received methadone treatment while incarcerated reported less heroin use, other opiate use, and injection drug use at 6-months post-release compared to those who were referred to methadone treatment after release. Dole et al. (1969) found that those assigned to methadone during incarceration were less likely to relapse and continue using once released compared to a control group, however results did not include hypothesis testing and suitable analyses. Another study found that engaging in community-based treatment post-release was associated with lower drug use (Magura et al., 1993). One study found no significant differences in opioid-positive urine screens at 12- and 24- months post-release between those receiving interim methadone during incarceration and those in treatment-as-usual (Schwartz et al., 2020; Schwartz et al., 2021).

#### Buprenorphine

Gordon et al. (2017) found no differences in post-release self-reported opioid use or opioid-positive urine screens between participants who received buprenorphine while incarcerated and a counseling-only control group. Zaller et al. (2013) found that none of the participants who initiated buprenorphine/naloxone while incarcerated reported any opioid use or injection drug use during the 6-month follow-up period, although there was not a significant difference between those who initiated buprenorphine/naloxone prior to release and those who initiated post-release. Magura et al. (2009) found no differences in post-release opioid use between participants who received buprenorphine treatment while incarcerated and a methadone treatment comparison group.

#### Naltrexone

Studies that examined the effects of starting XR-NTX while incarcerated on opioid use post-release were generally positive, at least during short-term follow-up periods after release. Lee et al. (2015) found that rates of opioid use at one-month post-release were lower among participants who received XR-NTX while incarcerated compared to those who did not receive medication. Friedmann et al. (2018) found that those who received XR-NTX prior to release had more days of confirmed abstinence during the first month post-release than those who started after release, and time to relapse was longer on average for the pre-release group as well. Gordon et al. (2015) studied participants who had their first XR-NTX injection prior to release, comparing those who received at least 6 injections to those who received fewer, finding that those who received at least 6 were significantly less likely to use opioids (assessed via urine and self-report) than those who received fewer. Studies by Farabee et al. (2020) and Woody et al. (2021) found no significant differences in post-release opioid use among participants who received XR-NTX compared to comparison groups who initiated XR-NTX after release or received enhanced treatment-as-usual.

#### Levo-alpha Acetylmethadol

One study examined the effect of LAAM treatment during incarceration on post-release opioid use. Kinlock et al. (2005) found no differences in post-release opioid use during a 9-month follow-up period between participants who received LAAM while incarcerated and those who did not.

### Opioid-related overdoses

#### Methadone

Several studies examined the effect of methadone treatment during incarceration on post-release opioid-related overdoses. Brinkley-Rubinstein et al. (2018) found that participants who received methadone treatment while incarcerated were significantly less likely to experience a non-fatal overdose during the 12-month follow-up period. A retrospective case-control study with a large sample found that continuation of methadone during incarceration was associated with significantly lower rates of non-fatal overdoses post-release when compared to a control group who endured forced taper and withdrawal upon incarceration (Haas et al., 2021). Kinlock et al. (2009) observed that four opioid-related overdose deaths occurred during the 12-month post-release follow-up period among participants enrolled in counseling-only as compared to zero overdose deaths among those enrolled in methadone during incarceration. During their study which included a 24-month post-release follow-up period, Schwartz et al. (2021) noted that none of the 9 opioid-related deaths among their sample of 225 participants occurred during methadone treatment. Furthermore, of the 87 non-fatal opioid overdoses recorded during the 24-month follow-up period, only 4.6% occurred during methadone treatment.

#### Buprenorphine

Zaller et al. (2013) found that although the number of non-fatal overdoses experienced was small, none of the 11 participants who initiated buprenorphine while incarcerated reported an overdose during the 9-month follow-up period compared to 3 of the 25 participants who initiated buprenorphine post-release.

#### Naltrexone

Studies generally indicated higher overdose risk associated with abstinence-based treatment models involving XR-NTX, with all overdoses occurring after stopping XR-NTX. In a study by Lincoln et al. (2018) comparing pre-release vs. post-release initiation of XR-NTX, there were three overdose deaths, all in the pre-release group after they stopped XR-NTX. In the Woody et al. (2021) study, four died from overdoses after stopping XR-NTX including one who had started XR-NTX pre-release and three who started post-release. Both Lee et al. (2015) and Gordon et al. (2015) reported that no overdoses occurred during their studies examining XR-NTX.

#### All medications

Green et al. (2018) examined state mortality records before and after the Rhode Island Department of Corrections’ statewide implementation of MOUDs. They found that there was a 60.5% reduction in mortality amongst recently incarcerated individuals due to overdose deaths after the implementation of the state-wide program which continued MOUDs (methadone, buprenorphine, and naltrexone) during incarceration.

### Criminal involvement

#### Methadone

The studies which examined the effect of methadone treatment on criminal involvement outcomes showed positive results, either indicating less risk of criminal involvement or no effect. Brinkley-Rubinstein et al. (2018) noted that a lower proportion of participants who continued methadone upon incarceration were re-incarcerated after release compared to participants in the forced withdrawal group, however the difference was not significant. Dole et al. (1969) found that those assigned to methadone during incarceration were less likely to have additional convictions compared to a control group during their 7-to-10-month follow-up period, however results did not include hypothesis testing nor suitable analyses. During an 11-year period of monitoring, 80% of KEEP participants returned to Rikers Island only once or twice (Tomasino et al., 2001). Moore et al. (2018) found that participants who received methadone from the same provider prior to, during, and after incarceration had a reduced risk of re-arrest, new charges, and re-incarceration, and Magura et al. (1993) found that being in treatment at follow-up was associated with lower criminal involvement. Another study found no differences in recidivism in participants who received methadone treatment while incarcerated compared to counseling-only comparison groups at 1-, 6-, and 12-months post-release (Gordon et al., 2008; Kinlock et al., 2007; Kinlock et al., 2009). Similarly, McKenzie et al. (2012) did not find any statistically significant differences in arrest history or re-incarceration between methadone treatment and control groups. Haas et al. (2021) found that continuing methadone treatment while in jail had no apparent effect on the rate of recidivism compared to those who were forced to taper and withdraw. Another study found no significant differences between groups in self-reported criminal behavior or number of reported arrests at 12- and 24-months post-release (Schwartz et al., 2020; Schwartz et al., 2021). One study that focused on the impact of methadone treatment in jail on subsequent arrests found no significant differences between groups for re-arrest at 12-months post-release (Kelly et al., 2020).

#### Buprenorphine

The studies which examined the effect of buprenorphine on criminal involvement outcomes were also positive, either indicating less risk of criminal involvement or no effect. Evans et al. (2022) found that fewer participants who received buprenorphine while incarcerated recidivated compared to a control group. Magura et al. (2009) found no differences between participants who received buprenorphine treatment while incarcerated and a methadone comparison group in self-reported re-arrests, self-reported severity of crime, or recidivism during a 3-month post-release follow-up period. A study conducted by Gordon et al. (2018) that focused on arrest outcomes found no significant differences between groups in the proportion of participants arrested, number of arrests, time to first arrest, or severity of charges. Another study conducted by Gordon et al. (2017) found no significant differences at 12-months post-release in self-reported days of crime between those that initiated buprenorphine in prison compared to those who initiated after release. Zaller et al. (2013) noted that while self-reported re-arrests during the follow-up period were generally low, all re-arrests occurred in the group that initiated buprenorphine post-release and none occurred among those who initiated buprenorphine while incarcerated. Additionally, participants who were not continuously receiving buprenorphine treatment during the follow-up period were more likely to report re-arrest at 6-months post-release (Zaller et al., 2013).

#### Naltrexone

Regarding the effect of XR-NTX on criminal involvement, Lee et al. (2015) found no significant differences in rates of recidivism between randomly assigned groups, but it should be noted that participants who completed at least two injections of XR-NTX post-release had significantly fewer rearrests compared to participants who only had one injection post-release and the no-medication control group. Gordon et al. (2015) found no differences in post-release criminal involvement between those who received at least six XR-NTX injections and those who received fewer. Farabee et al. (2020) also found no significant differences in post-release criminal involvement post-release between conditions involving XR-NTX and a no-medication condition.

#### Levacetylmethadol

Kinlock et al. (2005) found no significant differences in criminal activity between participants who received LAAM treatment while incarcerated compared to community treatment referral.

### Quality and limitations of included studies

Several limitations of the included studies should be considered when interpreting their results and implications for future policy and research. Many of the included studies had samples comprised of only or mostly males (Brinkley-Rubinstein et al., 2018; Gordon et al., 2008; Haas et al., 2021; Kinlock et al., 2005; Kinlock et al., 2007; Kinlock et al., 2009; Lee et al., 2015; Moore et al., 2018) and most studies were conducted at a single site. So, the results discussed here may not be generalizable to the entire population of people who are incarcerated with OUD and should be applied with caution pending larger scale replications.

Ten of the included studies were non-experimental (see Table 1) which limits causal inferences from those studies’ results. However, the studies by Green et al. (2018) and Haas et al. (2021) had large sample sizes compared to the other reviewed studies and yielded some of the most convincing results regarding the effects of MOUDs on post-release opioid-related overdoses which occur at relatively low rates compared to other outcomes, thus necessitating larger sample sizes to detect treatment effects. It should also be noted that while many of the included studies set out to conduct true experiments, many had issues with consistency of randomization among samples used for analyses due to attrition during incarceration (e.g. voluntary withdrawal from medication, transfer to another correctional institute) and loss to follow-up often due to reincarceration (e.g., Gordon et al., 2008; Kelly et al., 2020; Kinlock et al., 2009; Lee et al., 2015).

Many studies, especially those that examined XR-NTX, had difficulty accessing participants for follow-up assessments due to reincarceration, housing instability, and other reasons. While biological confirmation of substance use is always preferred, Woody et al. (2021) were able to supplement their follow-up assessments by collecting data over the phone and via text messages. Many studies did not report providing incentives, and doing so may have increased response rates especially during longer follow-up periods.

## Discussion

This article aimed to review current evidence that examined post-release outcomes associated with the use of MOUDs in prison and jail settings. Results showed compelling evidence supporting the use of MOUDs for currently incarcerated populations, with almost all studies showing that providing MOUDs during incarceration increases post-release community-based treatment engagement. Findings indicate that forced tapering and withdrawal during incarceration can lead to an increased risk of opioid-related death upon release into the community. There is strong evidence to support the adoption of MOUDs, especially methadone, in correctional institutes due to their association with decreased opioid use, injection drug use, and overdoses after release from incarceration. Examinations of post-release criminal involvement were also favorable, with studies showing that providing MOUDs during incarceration is either associated with lower criminal involvement post-release or no effect. While research among other populations has shown that the effectiveness of buprenorphine is comparable to methadone, more research on the adoption and implementation of buprenorphine in correctional settings is still needed.

Results indicated support for the effectiveness of methadone in correctional facilities to increase community treatment engagement, reduce opioid use, and reduce injection drug use post-release. These findings have important implications given the increased overdose risk that people with OUD experience when released from incarceration (Merrall et al., 2010). Decreases in injection drug use reduce the risk and spread of infectious diseases like HIV and hepatitis C (Wejnert et al., 2012; Thorpe et al., 2000). Recently there have been rapid increases in the use of illicitly manufactured fentanyls (IMFs) and related overdoses in the U.S. (Centers for Disease Control and Prevention, 2021). Due to it being a full agonist without a ceiling effect, methadone is likely the best MOUD for individuals entering jails and prisons dependent on high doses of IMFs and other opioids (Bromley et al., 2021).

Results related to post-release criminal involvement were also supportive of providing MOUDs while incarcerated. There was no evidence that providing MOUDs during incarceration leads to increases in criminal involvement post-release, and it may be associated with reductions in criminality. Outcomes related to criminal involvement tended to be either lower among those who received MOUDs while incarcerated or roughly the same compared to those who did not. Several studies reported non-significant findings that were nonetheless favorable for providing MOUDs during incarceration. For example, Gordon et al. (2018) observed that mean number of arrests were lower and mean days until rearrest were higher among those who initiated buprenorphine while incarcerated compared to those who started in the community after being release. Similarly, Lee et al. (2015) found that re-incarceration rates were lower among those who started XR-NTX prior to release compared to those who did not, although not statistically significant. Studies that analyzed criminal involvement among those who did engage in post-release treatment showed reductions in re-arrest, new charges, and re-incarceration (Lee et al., 2015; Moore et al., 2018). Results showed substantial evidence for the effectiveness of MOUDs while incarcerated in increasing community-based treatment engagement post-release, therefore, these findings suggest that criminal involvement may be lowered among people who continue or initiate MOUDs while incarcerated and then continue to engage in community-based MOUDs after release.

Because XR-NTX is an antagonist and has no potential for misuse, it tends to be more acceptable to jail and prison administrators and thus may face fewer barriers to adoption. However, similar to research with other populations, results from the studies reviewed here indicate that XR-NTX is less effective than agonist treatments that are initiated during incarceration. Given that XR-NTX is the newest MOUD approved by the FDA, people with OUD often lack knowledge about it. Velasquez et al. (2019) found that most who were offered XR-NTX while incarcerated had never heard of it and were skeptical of its blockade effectiveness and its ability to reduce cravings. This lack of knowledge and skepticism towards XR-NTX’s effectiveness may lead to increased opioid use post-release that fades once individuals experience for themselves how XR-NTX works. Efforts to increase knowledge and exposure to XR-NTX among people with OUD are needed to increase its acceptability.

Providing XR-NTX shortly before release from incarceration may be an effective way to prevent relapse during the first 1–2 months after release, however this appears to accompany an increased risk of fatal overdose. Despite its questionable effectiveness and potential for increasing risk of overdose after stopping treatment, XR-NTX may be an important option for those who are not interested in agonist treatments. Studies examining XR-NTX often specifically recruited participants who were not interested in agonist treatments. Velasquez et al. (2019) found that those who opted to take XR-NTX often did so because they were not interested in agonist treatments for personal reasons, due to limited access to agonist treatments, or due to stigma associated with agonist treatments.

More research on continuing or initiating buprenorphine during incarceration is needed, especially given the fact that buprenorphine is more accessible to many participants who will re-enter rural and suburban communities. Buprenorphine is also more attractive to many individuals seeking recovery in the community since it is offered in office-based settings in contrast to methadone which is only offered at federally certified opioid treatment programs which require frequent visits and are highly stigmatized. Perhaps the office-based nature of buprenorphine treatment contributed to Magura et al. (2009) finding those who started buprenorphine while incarcerated were more likely to report to community treatment than those who started methadone prior to release. Velasquez et al. (2019) found that regulations, misinformation, and stigma associated with methadone affected treatment adherence, with some reporting that they took methadone while incarcerated only because they wanted to prevent withdrawals with the intention of tapering off and resuming illegal opioid use once they were released.

While research indicated that initiating or continuing MOUDs during incarceration led to increases in community treatment engagement post-release, attrition from community treatment was often high during longer follow-up periods, likely due to the many barriers that this population often encounters while seeking to engage in recovery services. Additional research should study barriers to accessing MOUDs that people involved in the criminal legal system with OUD may face post-release, particularly among women who are an understudied segment of this population.

Some studies were conducted in large prison systems while others were conducted in jails where sentences are generally shorter, allowing less time to initiate or continue MOUDs prior to release. Studies conducted in jails also experienced more difficulties with maintaining randomization counts due to participants being release earlier or later than expected and being transferred to other facilities. Many of the jails where studies were conducted were in large metropolitan locations and rival prisons in other parts of the country with regards to their capacity. In fact, nearly all of the studies were conducted in the densely populated Northeastern and Mid-Atlantic regions of the United States. More research is needed on the adoption and implementation of MOUDs in jails and prisons in other parts of the country, especially predominantly rural areas. It is not always the case that incarcerated individuals will be released into communities where there is sufficient access to MOUDs, particularly for those who initiate or continue methadone which tends to be absent from many rural areas. Buprenorphine and XR-NTX may be much better options for jails and prisons in predominantly rural regions since individuals continuing treatment in the community are more likely to have access to these MOUDs.

All people with OUD deserve access to evidence-based treatments whether incarcerated or not, however those who are already receiving MOUDs at the time of their arrest are an especially important group to provide MOUDs to throughout incarceration. In fact, the Department of Justice has issued guidance for the protections of people with OUD stating that those who are engaging in recovery by taking MOUDs at the time of their incarceration have a right to access MOUDs during incarceration and denying them access violates the Americans with Disabilities Act (U.S. Department of Justice, 2022). Most correctional systems, especially local jails, do not offer any type of MOUD during incarceration. The relatively few correctional systems that do provide MOUDs to those who are incarcerated only do so during the weeks or monthly leading up to release. For individuals who are incarcerated while currently taking methadone, buprenorphine, or illegal opioids in the community, forced withdrawal is unfortunately the norm across the United States. Transitioning from the community to a jail or prison is highly stressful and made exponentially more difficult for those who are forced to withdraw from opioids. Given the recent DOJ guidance and the inhumane nature of forced withdrawal from opioids, correctional systems must adopt and implement MOUDs not only for those who are about to be released but for all people with OUD throughout the time they are incarcerated. More research on continuing methadone and buprenorphine upon incarceration is also needed to examine post-release outcomes related to this policy.

## Limitations of this review

This review is limited by the fact that it is based on a search of only peer-reviewed journals. There may be dissertations or studies in grey literature that were not reviewed. Due to the authors being fluent only in the English language, we did not include articles published in other languages which is a limitation. Also, we only included studies that were conducted in the U.S. because of the unique context of the U.S. criminal legal system, however it is important to acknowledge this as a limitation. The generalizability of the results reported on in this review are limited by the inclusion of only studies conducted in the U.S. and by fact that many of the included studies were concentrated in certain geographical regions, ones that include many large metro areas.

The results and implications of this review are limited by the fact that only a narrative review was conducted. Engaging in meta-analytic methods with the included studies was not feasible due to the heterogeneity of interventions and methods implemented across studies. Four different medications were represented across studies, and correctional facilities varied considerably in how these medications were implemented (i.e., length of medication treatment prior to release). Comparison groups also varied greatly with few studies including a true control group, making it difficult to isolate the impact of MOUDs during incarceration.

## Conclusions

This review provides evidence to support the adoption and implementation of MOUDs in U.S. jails and prisons in order to continue or initiate these evidence-based treatments among incarcerated individuals with OUD. Based on the reviewed studies, there are major implications for correctional facilities nationally. All forms of MOUDs should be available to those who are incarcerated in the United States, either to initiate these evidence-based treatments for OUD while incarcerated or to continue treatment that began in the community and prevent forced withdrawal. Findings suggest that providing MOUDs to people who are incarcerated increases the likelihood that they will engage in treatment post-release while decreasing their risk of relapse post-release, all without increasing criminal involvement. Recidivism and reincarceration are costly to society and these results suggest that implementation of MOUDs in correctional institutions is not only feasible and humane, but it is also an important component of providing those with OUD who are incarcerated with the best chance of successful reintegration into communities upon release.

## Data Availability

Not applicable.
